# Social Cognition in Down Syndrome: Face Tuning in Face-Like Non-Face Images

**DOI:** 10.3389/fpsyg.2018.02583

**Published:** 2018-12-18

**Authors:** Marina A. Pavlova, Jessica Galli, Federica Pagani, Serena Micheletti, Michele Guerreschi, Alexander N. Sokolov, Andreas J. Fallgatter, Elisa M. Fazzi

**Affiliations:** ^1^Department of Psychiatry and Psychotherapy, Medical School and University Hospital, Eberhard Karls University of Tübingen, Tübingen, Germany; ^2^Department of Clinical and Experimental Sciences, University of Brescia, Brescia, Italy; ^3^Unit of Child and Adolescent Neurology and Psychiatry, ASST Spedali Civili di Brescia, Brescia, Italy; ^4^Women’s Health Research Institute, Department of Women’s Health, Medical School and University Hospital, Eberhard Karls University of Tübingen, Tübingen, Germany; ^5^LEAD Graduate School and Research Network, Eberhard Karls University of Tübingen, Tübingen, Germany

**Keywords:** Down syndrome, face resemblance, Face-n-Food paradigm, visual social cognition, neurodevelopmental disorders

## Abstract

Individuals with Down syndrome (DS) are widely believed to possess considerable socialization strengths. However, the findings on social cognition capabilities are controversial. In the present study, we investigated whether individuals with DS exhibit shortage in face tuning, one of the indispensable components of social cognition. For this purpose, we implemented a recently developed Face-n-Food paradigm with food-plate images composed of food ingredients such as fruits and vegetables. The key benefit of such face-like non-face images is that single elements do not facilitate face processing. In a spontaneous recognition task, 25 children with DS aged 9 to 18 years were presented with a set of Face-n-Food images bordering on the Giuseppe Arcimboldo style. The set of images was administered in a predetermined order from the least to most resembling a face. In DS individuals, thresholds for recognition of the Face-n-Food images as a face were drastically higher as compared not only with typically developing controls, but also with individuals with autistic spectrum disorders and Williams-Beuren syndrome. This outcome represents a significant step toward better conceptualization of the visual social world in DS and neurodevelopmental disorders in general.

## Introduction

Down syndrome (DS) is a set of cognitive and physical conditions that result from having an extra copy of chromosome 21. This is one of the most common sporadic genetic disorders with prevalence generally estimated around 1 per 1,000 live births: 1.447 in the United States in 2004–2006 (Parker et al., 2010) or more recently in the Netherlands, about 1.11 (de Graaf et al., 2017b). Latest analyses show that the number of people living with DS has steadily increased from 1950 until 2010 in several states of the United States, and population prevalence would have been even higher without DS-related elective terminations (de Graaf et al., 2016, 2017a). There is also some evidence for an increase in DS in Europe after the Chernobyl reactor accident (Sperling et al., 2012). The decreasing age of mothers giving birth to infants with DS as well as the increasing number of young parents (less than 35 years) of DS infants is also alerting (Verma and Huq, 1987).

Cognitive and motor development is delayed in individuals with DS. Common physical traits include craniofacial and musculoskeletal abnormalities, short stature, and hypermobility of the joints (Frith and Frith, 1974; Hickey et al., 2012). Children with DS have intelligence quotient (IQ) levels ranging between 36 and 107, and declining with age (Roizen and Patterson, 2003). The specificity of the DS profile includes relatively strong social skills, weak expressive language, and rather poor motor coordination. In DS, the considerable dissociation between boosted social motivation and social adeptness (having friends, being involved in social networks) may point to some difficulties in social cognition (Iarocci et al., 2008; Watt et al., 2010; Goldman et al., 2018). Children with DS (aged 10 to 18 years) exhibit more difficulties in judging, identifying and reasoning about transgression of social rules (Barisnikov and Lejeune, 2018). Adapted daily life social participation of DS individuals is of tremendous value for their quality of life, and therefore investigation of visual social cognition in this population is of high social and clinical relevance.

Faces and bodies provide us with a wealth of socially relevant information. Body language reading and face processing are indispensable components of non-verbal communication and interpersonal interaction constituting a core of social competence (de Gelder, 2009; Tamietto et al., 2009; Kret and de Gelder, 2012; Pavlova, 2012, 2017a,b; Pelphrey et al., 2014; Negro et al., 2015; Di Giorgio et al., 2017; Pavlova et al., 2017a). It has been suggested that social cognitive abilities and body motion processing are tightly linked and, therefore, performance on body motion tasks may serve a hallmark of social cognition (Pavlova, 2012): Individuals with such neurodevelopmental conditions as autistic spectrum disorders (ASD), Williams-Beuren syndrome (WS), DS, and survivors of premature birth who exhibit aberrant body motion processing, are also likely to be compromised on daily life social perception. In agreement with this assumption, newborn human infants and newly-hatched chicks appear to be predisposed to point-light biological motion (when body motion is represented by a set of lights placed on the main joints of an otherwise invisible actor) and face-like configurations. Such social predispositions are impaired in newborns at high risk of autism (Di Giorgio et al., 2016, 2017).

In accordance with popular wisdom that DS individuals possess socialization strengths (e.g., Fidler et al., 2006; Hippolyte et al., 2010), children with DS aged 8–15 years are reported to be able reliably recognize emotions (such as happiness, madness, and scare) from impoverished point-light displays of an experienced female point-light dancer, but fail in recognition of sadness (Virji-Babul et al., 2006). Yet they are unsuccessful in discrimination of typical and atypical (represented by individuals with cerebral palsy and DS) point-light gaits. The same study reports that children with DS reliably discriminate between point-light persons and objects, though their performance level is lower than in typically developing (TD) controls. In accord with this outcome, more recent data indicates that DS individuals are fairly capable of discriminating (though less accurate in discrimination) between canonical and scrambled versions of point-light body motion, and exhibit ceiling performance level in detecting direction (left- or rightward) of a canonical point-light figure and scrambled displays moving with translational component across the screen (Riddell et al., 2017). Magnetoencephalography (MEG) shows that in adults with DS, observation of body motion elicits the pattern of activation of the cortical network that is similar to TD controls but more scattered (Virji-Babul et al., 2010). In general, it appears that individuals with DS can reveal social information from body motion, but this ability is compromised and modulated by the emotional context of actions.

Faces represent valuable signals for effective interpersonal interaction (Kret and de Gelder, 2012). In agreement with widespread beliefs about friendliness and social accessibility, adults with DS exhibit a tendency to judge emotional face expressions more positively than controls (Hippolyte et al., 2008) or did not show any specific deficits in recognizing emotional expressions in static and, in particular, dynamic faces (e.g., Carvajal et al., 2012; Channell et al., 2014; Pochon et al., 2017). On an emotion attribution task with faces, adults with DS exhibit difficulties with sad face expressions (Hippolyte et al., 2008) similar to poor recognition of sad point-light dance (Virji-Babul et al., 2006; see above). By contrast, other studies report that individuals with DS have some troubles in facial affect recognition, in particular, in interpreting fear and surprise (Wishart and Pitcairn, 2000; Wishart, 2001; Williams et al., 2005; Wishart et al., 2007; Cebula and Wishart, 2008; Iarocci et al., 2008; Cebula et al., 2017). In DS, when happy and angry faces are used as either positive or negative feedback on a task, angry faces do not inhibit subsequent performance (Goldman et al., 2018).

Little research has been carried out on face processing and sensitivity in DS. Although children aged 8 to 14 years are slower than TD controls at identity-matching tasks, their accuracy is not significantly different (Wishart and Pitcairn, 2000). Children with DS are poor at processing features in the Benton facial recognition test, and discriminate facial features better when presented in whole faces than in isolation (unlike the other groups with neurodevelopmental disorders such as children with ASD or WS). Apparently, the clue of a whole face helps them to recognize face features (Annaz et al., 2009). Performance of DS children is poor in a modified version of the ‘Jane faces’ task (that serves to disentangle featural and configural face processing) with better accuracy on featural (with replaced eyes or mouth) over configural (with changes in distance between eyes on the face) trials (Mondloch et al., 2002). On this task, DS children exhibit the lack of inversion effect and only very slight improvement across age (Dimitriou et al., 2015).

**FIGURE 1:**
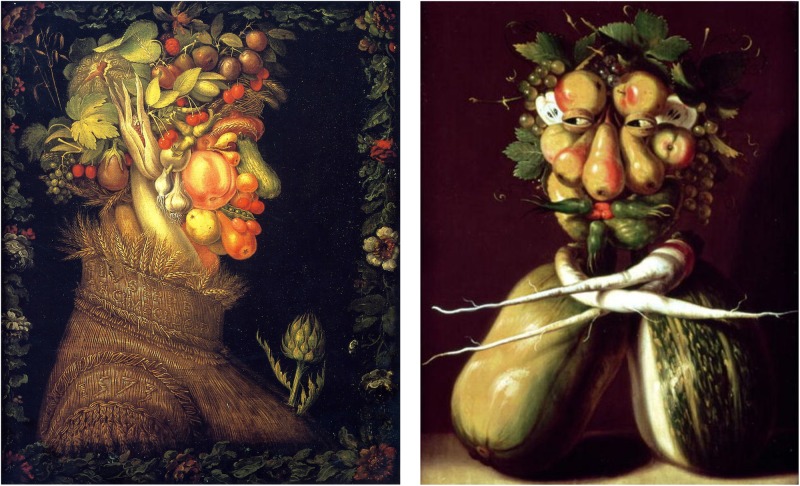
Examples of the Giuseppe Arcimboldo style. ‘Summer’ (left) and ‘Whimsical Portrait’ (right) by Guiseppe Arcimboldo (1526–1593), an Italian painter best known for creating fascinating, often grotesque and allegoric, imaginative portraits composed of fruits, vegetables, plants, tree roots, flowers, and even books and human/animal bodies (https://commons.wikimedia.org/wiki/Giuseppe_Arcimboldo; https://www.bridgemanimages.com/; public domain).

**FIGURE 2:**
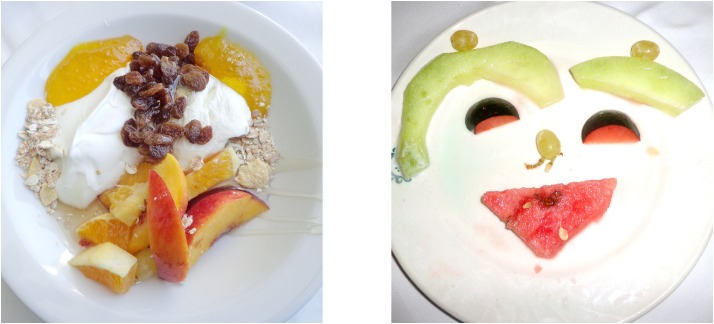
Examples of Face-n-Food images. The least resembling face (left panel) and most resembling face (right panel) images from the Face-n-Food task (from Pavlova et al., 2015). © The Creative Commons Attribution License [CC BY 4.0].

It remains unclear whether individuals with DS experience shortage in face tuning. Recently, a new experimental tool had been proposed for studying face tuning (Pavlova et al., 2015, 2016a,b, 2017b, 2018): a set of food-plate images composed of food ingredients (fruits, vegetables, sausages; see Figures 1, 2) was created in a manner slightly bordering on the style of Giuseppe Arcimboldo (1526–1593), an Italian painter best known for fascinating (very often grotesque and allegoric) imaginative portraits composed entirely of fruits, vegetables, plants, and flowers. The primary advantage of Face-n-Food images is that single components (food ingredients) do not explicitly trigger face processing. In addition, in the Face-n-Food task, face tuning occurs spontaneously without being explicitly cued by face elements such as eyes or brows. In this explorative study, we intended to clarify whether DS individuals exhibit scarcity in processing face-like Face-n-Food stimuli.

## Materials and Methods

### Participants

Twenty five individuals with DS (6 females, 19 males) were enrolled in the study. They were recruited at the Unit of Child and Adolescent Neurology and Psychiatry of Asst Spedali Civili (Civil Hospital) of Brescia, Italy. All DS children had previously been tested positively for trisomy of chromosome 21. Participants were aged 13.16 ± 2.14 years (mean ±*SD*; age range, 9 to 18 years). None of them had General IQ (GIQ) assessed by a standard procedure (WISC-IV, Wechsler Intelligence Scale for Children-IV, adapted to Italian population; Orsini et al., 2012) higher than 59: 14 participants had GIQ less than 40, and 11 participants had GIQ equal or higher than 40. The data of sixteen (1 female, 15 males) TD participants (aged 14.13 ± 2.14; age range, 11 to 17 years) recruited from the local community of Brescia, Italy, served as a control set; the data of this group was previously reported within the framework of the other study with autistic individuals (Pavlova et al., 2017b). No difference was found between DS and TD participants in respect to age [*t*(39) = 1.12, n.s.]. We did not match individuals with DS and TD controls in respect to IQ, as this matching can create unrepresentative groups for the respective populations, with either the group with neurodevelopmental disorder having a higher than representative IQ for this disorder or the control group having a lower than representative IQ (Dennis et al., 2009; Riddell et al., 2017). The data of DS individuals on subscales of the WISC-IV (Orsini et al., 2012) such as Perceptual Reasoning, Working Memory, Verbal Comprehension, and Processing Speed are summarized in Supplementary Table 1. Participants were run individually. All of them had normal or corrected-to-normal vision. None had previous experience with such images and tasks. The study was conducted in line with the Declaration of Helsinki and was approved by the local Ethics Committee of Asst Spedali Civili (Civil Hospital) of Brescia, Italy. Informed written consent was obtained from all participants or their care providers. Participation was voluntary, and the data was processed anonymously.

### The Face-n-Food Task

The Face-n-Food task was administered to participants. This task is described in detail elsewhere (Pavlova et al., 2015, 2016a,b, 2017b, 2018). For this task, a set of ten images was created that were composed of food ingredients (fruits, vegetables, sausages, etc.), and to different degree resembled faces. The images slightly border on the Giuseppe Arcimboldo style (Figures 1, 2). Participants were presented with the set of images, one by one, in the predetermined order from the least to most resembling a face (images 1 to 10). This order was determined in the previous study with TD volunteers (Pavlova et al., 2015). This fixed order had been used, because once seen as a face, Face-n-Food images are often processed with a strong face-dominating bias. On each trial, participants had to perform a spontaneous recognition task: they were asked to briefly describe what they saw. Their reports were recorded, and then analyzed by independent experts. For further data processing, the responses were coded as either non-face (0) or face (1) report. No immediate feedback was provided. To avoid time pressure that can potentially cause stress and negative emotional and physiological reactions blocking cognitive processes, there was no time limit on the task. With each participant, the testing procedure lasted no longer than 20–25 min.

## Results

As in earlier studies with TD individuals and participants with neurodevelopmental disorders such as WS or ASD (Pavlova et al., 2015, 2016a,b, 2017b, 2018), children with DS either described a food-plate image in terms of food composition (non-face response, 0) or as a face (face response, 1). When an image had been seen as a face, DS individuals (similar to TD individuals) often exhibited emotional reaction such as smiling and astonishment. On overall, however, DS individuals experienced much more troubles in spontaneous recognition of these images as a face. Strikingly, 14 DS participants out of 25 (56%) completely failed on the Face-n-Food task: they did not recognize even the images most resembling faces. Since no differences in performance (face response rate) of DS participants with GIQ less than 40 and GIQ equal or higher than 40 were found (Mann-Whitney test, *U* = 76, two-tailed, *p* > 0.05, n.s.), for further analyses, the data of these participants were pooled together.

We compared thresholds for face tuning (i.e., an average image number, on which face response was initially given on the Face-n-Food task) for DS individuals and TD controls. TD controls reported seeing a face for the first time on average on 3.56 ± 1.59 image (mean ±*SD*; median, 3.5; 95% confidence interval, CI, 2.78 to 4.34), and all of them perceived at least several images most resembling faces as a face. In DS individuals, spontaneous face tuning was drastically impaired: only 11 out of 25 participants reported seeing a face, giving the first face response on average on 7.55 ± 1.29 image (median, 7.0; 95% CI, 6.79 to 8.31). The difference in the face recognition thresholds between DS individuals and TD controls was highly significant (*Z* = -4.293, *p* < 0.0001, two-tailed; effect size, η^2^ = 0.45).

**FIGURE 3:**
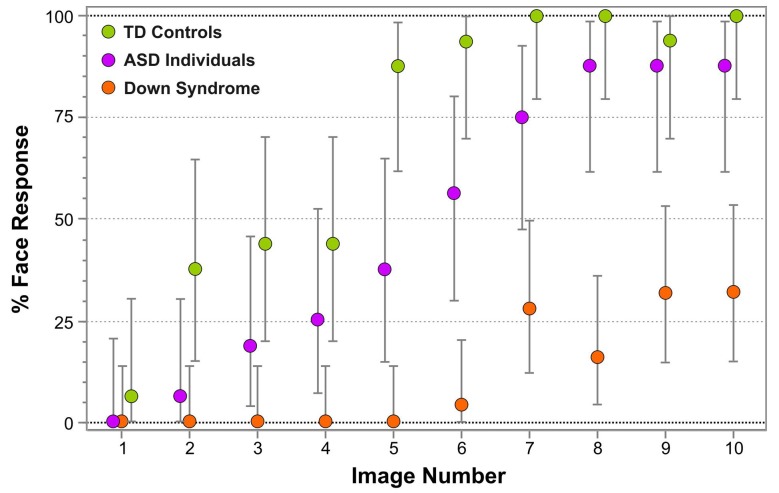
Percentage of face responses for each Face-n-Food image in individuals with Down syndrome (*orange*), typically developing (TD) controls (*green*) and individuals with autistic spectrum disorders (ASD; *violet*). The image number reflects its face resemblance (1 – the least recognizable through 10 – the most recognizable as a face). Vertical bars represent 95% confidence interval, CI. The data for TD participants and individuals with ASD had been reported earlier (Pavlova et al., 2017b). © The Creative Commons Attribution License [CC BY 4.0].

Figure 3 represents the percentage of face responses for each Face-n-Food image separately for DS individuals and TD controls. As seen from this Figure, individuals with DS much later reported seeing a face and gave overall much fewer face responses. As indicated by multiple stepwise nominal logistic regression analysis, the effect of group (TD vs. DS) is highly significant [χ^2^(1) = 117.5, *p* < 0.0001]. As compared to DS individuals, in TD controls the odds ratio to give the face response to each Face-n-Food image in the set is 139 (95% CI, 60 to 359; *p* < 0.0001). Remarkably, for the first five images that were less face resembling, DS individuals provided no face responses at all, whereas the TD group gave about 46% face responses already for images 3 and 4. Starting from the image 5, TD participants reached very fast the ceiling level of performance. By contrast, even on the images most resembling a face, DS individuals attained around 25–30% of face responses only. There is no significant Group × Image interaction [χ^2^(1) < 0.42, *p* > 0.52]: if the probabilities are transformed to log odds, the spontaneous face recognition curve is shifted down in DS individuals as compared to TD controls.

Figure 3 enables comparison of the data in individuals with DS not only with TD controls, but also with our earlier data in individuals with ASD (Pavlova et al., 2017b). Multiple stepwise nominal logistic regression analysis indicates that all three groups of participants significantly differ from one another [χ^2^(1) = 239.1, *p* < 0.0001]. In ASD individuals compared to DS individuals, the odds ratio to give the face response to each Face-n-Food image in the set is 20.8 (95% CI, 10.6 to 44.3; *p* < 0.0001). Again, there is no Group × Image interaction [χ^2^(2) < 0.79, *p* > 0.67]: on the logit scale, the curves for all three groups of participants are parallel.

In DS individuals, no correlation was found between their performance on the Face-n-Food task (face response rate) and the scores (standard points) on several subscales of the WISC-IV, which reflect specific cognitive abilities such as Verbal Comprehension (Spearman’s rho = 0.141, *p* = 0.512, n.s.), Working Memory (Spearman’s rho = -0.105, *p* = 0.617, n.s.), Perceptual Reasoning (Spearman’s rho = 0.143, *p* = 0.494, n.s.), and Processing Speed (Spearman’s rho = 0.256, *p* = 0.218, n.s.). This indicates that the poor performance on the Face-n-Food task in DS individuals is not substantially related to specific deficits in general cognitive abilities that may potentially affect task performance.

## Discussion

By implementing the recently created Face-n-Food task consisting of a set of food-plate images that comprised food ingredients such as fruits, vegetables, and sausages (Pavlova et al., 2015, 2016a,b, 2017b, 2018), we examined face tuning in children with DS. The key benefit of Face-n-Food images is that their single components do not explicitly trigger face processing. The outcome points to the extremely poor performance of DS individuals on the Face-n-Food task. Thresholds for spontaneous recognition of the Face-n-Food images as a face in DS individuals were drastically higher as compared not only with TD controls, but also with our earlier data in individuals with ASD (Pavlova et al., 2017b).

Bearing in mind that (i) individuals with DS exhibit primarily a global (holistic) style in face encoding (Annaz et al., 2009) and visuospatial processing in general (Bellugi et al., 1999a,b), and (ii) perceivers with global perceptual style appear to excel on recognition of faces in original Arcimboldo portraits (Boccia et al., 2014, 2015), the outcome in DS individuals is rather startling. Their extremely poor performance on the Face-n-Food task appears even more notable in view of enhanced social motivation and accessibility that characterize this population. The most plausible explanation for this data is that deficits on the Face-n-Food task originate from some difficulties of DS individuals in abstract/symbolic reasoning (e.g., Natsopoulo et al., 2002). Indeed, it might have been quite challenging to seeing a face in non-face images containing elements such as fruits and vegetables (instead of familiar eyes and a mouth) resembling a face by spatial arrangement of these elements solely. Just a few DS participants spontaneously recognized a face, i.e., perceived the single elements as a Gestalt, in images even with clear bonding between the elements. Notably, such images elicited a rather strong face impression not only in TD individuals, but also in children with ASD and WS (Pavlova et al., 2015, 2016a,b, 2017b, 2018). This upshot sheds light on the architecture of the specific visual social world in DS individuals.

In typical development, faces can be effortlessly seen in non-face images such as shadows, grilled toasts, ink blots, clouds or landscapes (Evritt, 2013). This phenomenon reflects sharp tuning to faces sometimes termed *face pareidolia*, when a socially vital face pattern is perceived where none exists, and often elicits emotional impressions (sympathetic face, angry face, silly face, etc.). Face pareidolia is widely used in commercial advertising and social media (smileys) as well as in driving regulations such as emoji road signs. Yet this is not a uniquely human phenomenon: the visual perception of facial features is driven by a broadly tuned face-detection mechanism that we share with other species such as the rhesus monkey (*Macaca mulatta*) (Taubert et al., 2017). The preference for both faces and face-like images is eliminated in monkey with bilateral amygdala lesions (Taubert et al., 2018): face pareidolia is likely to be reinforced already at the subcortical brain level. Neurons in the monkey superior colliculus preferentially filter face-like patterns at short latencies to allow for the rapid coarse facial processing and develop categorization of the stimuli at later latencies (presumably through feedback from upstream areas): Low-pass filtering of the images do not affect the responses, whereas image scrambling increases the responses at later latencies (Nguyen et al., 2014).

Human infants are well tuned to face-like images (e.g., Goren et al., 1975; Johnson et al., 1991; Kato and Mugitani, 2015) that points to some innate mechanisms for face sensitivity and a kind of social predisposition to faces (Di Giorgio et al., 2016). TD children aged 5 to 6 years exhibit orienting toward face-like stimuli (Shah et al., 2015). Studies with face-like non-face images show that TD children aged 24–60 months are more likely to direct their first fixation toward upright face-like objects than their peers with ASD (Guillon et al., 2016). Neurotypical population possesses an entire bias for seeing faces in Arcimboldo-like images: already 7–8 months old human infants prefer the Arcimboldo portraits over the same pictures inverted 180° in the image plane (Kobayashi et al., 2012). Patients with prosopagnosia (after right unilateral brain damage) and simultanagnosia are capable of seeing the Arcimboldo faces despite their evident ambiguity (Dalrymple et al., 2007; Busigny et al., 2010). In TD adults, ambiguous paintings are preferred over non-ambiguous more realistic ones: in watching abstract artwork, ambiguity is associated with greater pleasure and interest (Jakesch and Leder, 2009; Jakesch et al., 2013).

Converging lines of evidence from neuroimaging, neuropsychological and electrophysiological studies indicate that the subcortical face route (such as colliculo-pulvino-amygdalar pathway; Burra et al., 2017) provides a foundation for the cortical ‘social brain’ network (e.g., Gabay et al., 2014; Nguyen et al., 2014; Diano et al., 2017; Taubert et al., 2018). Alterations to this pathway might be decisive for some neurodevelopmental conditions characterized by impaired face processing and social cognition at large (Johnson, 2005).

The Face-n-Food test has already been implemented for testing other neurodevelopmental disorders, namely, ASD (Pavlova et al., 2017a) and WS (Pavlova et al., 2016a). As compared to individuals with DS, the sample of ASD individuals of the same age and cultural background is much less compromised on the Face-n-Food task despite well-known limitations of this population in social cognition (Figure 3). By contrast, although both DS and WS individuals possess some social strength or even a drive for social interaction (e.g., Bellugi et al., 2000) and a kind of face fascination, their tuning to coarse face information in face-like non-face images is extremely poor. Comparison of face tuning between DS and WS individuals [despite some differences between the samples in respect to gender, cultural background, which may affect performance on the Face-n-Food task (Pavlova et al., 2018), and age of participants (WS individuals in Pavlova et al., 2016a were aged 23.3 ± 10.6 years, age range 8 to 44 years, though chronological age is not a predictor of featural face decoding in WS and DS individuals; Annaz et al., 2009)] shows that DS participants experience much more troubles in spontaneous recognition of the Achimboldo-like images as a face. Although the thresholds of face tuning are comparable in these groups (on average, image 8.18 ± 1.47 in WS vs. 7.55 ± 1.29 in DS participants), there are substantial differences in their performance: (i) only 15% (three out of 20) WS participants but 56% (14 out of 25) DS participants completely failed on the task; (ii) as seen on Figure 3 of this paper and Figure 4 of Pavlova et al. (2016a), the recognition dynamics in two groups is remarkably different. Although both groups did not recognize the first five images as a face at all, face recognition in WS individuals continuously elevated resulting in the recognition rate above 85% for the most resembling a face image 10. By contrast, in DS individuals even this image elicited only about 30% face responses. This analysis suggests that face tuning scarcity may be of diverse origin in all these neurodevelopmental disorders.

Even in TD population, little research had been done on brain processing of face-like non-face images. The time course of the MEG response in the right ventral fusiform face area (FFA) during processing of faces and face-like non-face images is rather similar: the brain is likely to be hardwired to detect the presence of a face as quickly as possible, rather than to process face-like non-face images later on under top-down influence (Hadjikhani et al., 2009). In other words, it appears that face processing evoked by face-like non-face images is a relatively early process. This data suggests that deficits in perceiving face-like non-face images in DS individuals emerge already at early stages of brain processing. Yet the right superior temporal sulcus (STS) also differentiates between faces and face-like non-face images (Hadjikhani et al., 2009). Functional MRI indicates that the right FFA is active during perception of noise images containing components resembling a face: images even with the slightest face cues, i.e. only with coarse face information, are perceived as faces (Liu et al., 2014). Both event-related potential (ERP) and fMRI findings suggest that the interpretation of ambiguous stimuli depends upon processes similar to those elicited by familiar meaningful images (Voss et al., 2010, 2012): ‘To the brain, the vaguely Elvis-like potato chip can truly provide a substitute for the King himself’ (Voss et al., 2012; p. 2354), and be perceived not just as a tasty snack but as the embodiment of Elvis.

In TD adults, Arcimboldo portraits compared to classical Renaissance portraits and non-artistic face depictions (photographs) produce greater fMRI brain response in the occipito-temporal network underpinning face processing (including the FFA), bilateral superior and inferior parietal cortices, and the right inferior frontal gyrus (Boccia et al., 2015). When contrasted with the same upside-down paintings, Arcimboldo portraits elicit fMRI activation in the right FFA and posterior STS (Rossion et al., 2011), two indispensable parts of the social brain. In the right hemisphere, the face-sensitive N170 component of the ERP is the same in response to Arcimboldo portraits and real faces, whereas in the left hemisphere, the N170 amplitude is larger for faces (Caharel et al., 2013). This suggests the right hemispheric dominance in holistic processing of Arcimboldo-like images as faces.

It is essential to figure out whether face processing has a special status in DS individuals. In the present work, no correlation was found between their performance on the Face-n-Food task (face response rate) and scores on several subscales of the WISC-IV reflecting specific cognitive abilities such as Verbal Comprehension, Working Memory, Perceptual Reasoning, and Processing Speed. This suggests that the poor performance on the Face-n-Food task in DS individuals is not substantially related to some specific intellectual and cognitive disabilities, and rather points to aberrant face tuning as a most probable account for their performance over alternative explanations.

A skewed sex ratio (defined as the number of males divided by the number of females) with male prevalence has long been recognized in DS. Male prevalence of 1.23 is reported for England and Wales (Mutton et al., 1996), 1.27 for the Danish population (Nielsen et al., 1981), and about 1.36 for the New York area (Verma and Huq, 1987). The meta-analysis performed on 55 published studies also points to an overall sex ratio of 1.36, and considers genetic mechanisms responsible for male predominance in DS (Kovaleva, 2002). Gender specificity has been reported in tuning to the Face-n-Food images: females readily report the impression of a face while males still describe the same stimuli as food (Pavlova et al., 2015). Face resemblance is tightly linked with gender specific impression: images most resembling a face elicit more female-face responses in both female and male observers. Moreover, face resemblance is positively related to face likability, but this holds true only for female perceivers: images most resembling a face also appear more likable (Pavlova et al., 2016b). Most recent findings indicate that gender differences can be profoundly modulated by gender by culture interaction (Pavlova et al., 2018). The issues of (i) what is the nature of gender specificity in face processing and (ii) whether neural circuits underlying face tuning are sex specific remain open. The only study investigating sex differences in processing of face-like non-face images shows that the female brain is more sensitive to faces in non-face images: in women, the vertex positive potential (VPP) of electroencephalogram is the same for face depictions of real faces and face-like images, whereas in males it is lower for face-like objects (Proverbio and Galli, 2016). Moreover, in females, processing of face-like non-face images is associated with the activation of the brain areas involved in affective processing [the right STS, Brodmann area 22 (BA 22); posterior cingulate cortex, BA 22; and orbitofrontal cortex, BA 10], whereas in males, the activation of the occipito-parietal regions is dominant. As mostly male individuals with DS were enrolled in the present study, it is desirable to take a closer look at potential gender impact on face tuning in DS at both behavioral and brain levels. Such work will also contribute to better conceptualization of neurodevelopmental and neuropsychiatric conditions with a skewed sex ratio.

## Resume

The outcome of this work indicates that DS individuals experience substantial difficulties in perceiving faces in face-like non-face images. Thresholds for spontaneous recognition of the Face-n-Food images as a face in DS individuals were drastically higher as compared not only with TD controls, but also with ASD (Pavlova et al., 2017b) and WS (Pavlova et al., 2016a). Comparison of performance between all three samples of neurodevelopmental disorders sheds light on the nature of face tuning scarcity in DS. Deficits in face tuning of individuals with DS, WS and ASD appears to be of diverse origin. Clarification of the precise sources of these aberrations including differences in dynamic topography of the brain networks underwriting face tuning represents a major challenge for future work.

## Author Contributions

MP conceived and designed the study. JG, FP, and SM performed the experiments. MP, JG, and AS analyzed the data. MP, MG, AS, AF, and EF contributed reagents, materials, and analysis tools. MP, AS, and EF wrote the paper. MP and EF supervised the whole project.

## Conflict of Interest Statement

The authors declare that the research was conducted in the absence of any commercial or financial relationships that could be construed as a potential conflict of interest.
