# Supplementary material for: Social Cognition in Down Syndrome: Face Tuning in Face-Like Non-Face Images
Source: Front Psychol. 2018 Dec 18;9:2583. doi: 10.3389/fpsyg.2018.02583 (PMC6305370; doi:10.3389/fpsyg.2018.02583)
Supplement: Supplementary file 1 [file Table_1.DOCX]

**Supplementary Table 1.**

The scores (standard points) of DS individuals on the WISC-IV subscales.

| **Participant Code** | **Working Memory** | **Perceptual Reasoning** | **Processing Speed** | **Verbal Comprehension** |
| --- | --- | --- | --- | --- |
| **P1** | 46 | 41 | 47 | 56 |
| **P2** | 46 | 41 | 47 | 46 |
| **P3** | 46 | 56 | 62 | 50 |
| **P4** | 49 | 50 | 50 | 64 |
| **P5** | 46 | 41 | 47 | 46 |
| **P6** | 46 | 41 | 47 | 46 |
| **P7** | 46 | 41 | 47 | 46 |
| **P8** | 70 | 61 | 68 | 76 |
| **P9** | 46 | 43 | 47 | 46 |
| **P10** | 46 | 41 | 47 | <46 |
| **P11** | 46 | 52 | 76 | 46 |
| **P12** | 46 | 41 | 47 | 46 |
| **P13** | 46 | 45 | 59 | 48 |
| **P14** | 49 | 41 | 47 | 46 |
| **P15** | 46 | 41 | 47 | 46 |
| **P16** | 46 | 41 | 50 | 52 |
| **P17** | 46 | 41 | 47 | 46 |
| **P18** | 49 | 61 | 47 | 58 |
| **P19** | 52 | 50 | 68 | 62 |
| **P20** | 46 | 41 | 47 | 46 |
| **P21** | 49 | 58 | 62 | 76 |
| **P22** | 61 | 58 | 59 | 66 |
| **P23** | 49 | 58 | 50 | 62 |
| **P24** | 55 | 76 | 68 | 66 |
| **P25** | 49 | 67 | 71 | 50 |
